# Decision making and benefit analysis of closed-loop remanufacturing supply chain considering government subsidies

**DOI:** 10.1016/j.heliyon.2024.e38487

**Published:** 2024-09-26

**Authors:** Peng Wan, Zhiyuan Xie

**Affiliations:** School of Management Engineering, Qingdao University of Technology, Qingdao, 66525, China

**Keywords:** Circular economy, Game theory, Social welfare, Recycling, Investment

## Abstract

Remanufacturing closed-loop supply chain can effectively deal with the deterioration of environmental resources and the limited supply of resources as a part of circular economy. Government subsidies are an important factor to promote its development. In this paper, we consider a two-stage remanufacturing closed-loop supply chain, where the retailer is responsible for recycling, and the manufacturer will innovate in remanufacturing technology based on the extended producer responsibility system. The study constructs a Stackelberg game model with the manufacturer as a leader, and compares the different impacts of the two subsidy strategies on supply chain decisions (i.e., subsidies for investment in remanufacturing technology innovation and subsidies for the cost of remanufactured products). Numerical analyses are conducted to visualize the effects of subsidies on the decision-making of closed-loop supply chain members, environmental benefits, and social welfare. The findings indicate that when the level of subsidy is low, government subsidies for investment in remanufacturing technology innovation can accelerate the upgrading of remanufacturing technology, leading to higher environmental benefits and social welfare compared to subsidising the cost of remanufactured products. The results show that when the level of subsidy is high, government subsidies for remanufactured product costs, while yielding greater environmental benefits and social welfare compared to subsidies for investment in remanufacturing technology innovation, have a lesser impact on the advancement of remanufacturing technology.

## 1 Introduction

With the deterioration of environmental resources and the waste of limited resources, traditional production methods and consumption patterns are not sustainable. The circular economy (CE) aims to reverse the current practices of rapid resource depletion and waste generation, while also creating value for products. It offers circular business models that emphasize recycling, remanufacturing and refurbishment in the design of eco-innovative products and services in the market [1]. CE thinking requires a comprehensive approach that seeks to overhaul economic and social systems in order to not only minimize humanity's impact on the environment, but also to establish a harmonious balance in human-nature relations [2]. Remanufacturing refers to the batch production process of specialized repair of damaged or end-of-life automotive parts, engineering machinery, machine tools and so on. When buyers dispose of end-of-life manufactured products, companies recycle the end-of-life products, giving the used products back their use value through remanufacturing. Remanufactured products can achieve the same quality and performance as the original new product. Remanufacturing not only extends the life of products from a manufacturing perspective, but also reduces the use of resources. Remanufacturing is an important part of the CE [3], and through recycling and remanufacturing, emissions could be reduced by 312–344 million tons of carbon dioxide equivalent by 2030 [4]. Recycling and remanufacturing are integral components of the closed-loop supply chain (CLSC), and are widely recognized as essential strategies for manufacturing companies seeking to realize economic and environmental benefits through recycling activities [5]. Therefore, companies worldwide are actively seeking ways to advance the value of remanufactured products and extend their lifespan through repairing old products. Technological change is a key factor in remanufacturing and requires more attention to achieve sustainable development [6].

Many countries have enacted legislation to promote waste collection and remanufacturing. This legislation can be categorized into two main types: take-back legislation and government subsidies. Take-back legislation requires manufacturers and remanufacturers to meet recycling or reuse targets, which are defined as a minimum percentage of new product sales. For instance, Japan's Specified Household Appliances Recycling Law (SHARL) sets a take-back target of 50–60 per cent of new products, while the Waste Electrical and Electronic Equipment (WEEE) Directive sets a reuse target of 4 per cent of new products. As for government subsidies, they are provided by the government to manufacturers and remanufacturers to incentivize them to recycle and remanufacture. The Scottish government applies £70 million in subsidy payments to encourage remanufacturing [7]. To promote recycling and remanufacturing in the supply chain, certain governments have implemented subsidy policies. For example, in 2010, the Liuyang Municipal Government in Hunan Province, China, offered a one-time subsidy equivalent to 20 % of the total investment in remanufacturing construction [8]. The government hopes to promote circular economy development and improve environmental and social welfare through subsidies, and this paper explores in more detail how government policies affect supply chain members to achieve their own purposes from the perspective of subsidies.

Promoting environmental protection and resource conservation to achieve sustainable development necessitates the collaborative efforts of members within the CLSC, as well as the active involvement of governmental bodies. In order to inspire remanufacture, the government can afford subsidies for remanufacturing costs and remanufacturing technology inputs. Let's consider the following questions:

(I) With the different subsidies provided by the government, how do manufacturers determine their level of remanufacturing technology?
(II) How do manufacturers and retailers determine their own pricing decisions under different subsidies?
(III) What is the effect of government subsidies on supply chain returns, environmental benefits, and social welfare in a closed-loop supply chain where retailers are responsible for recycling?

This study considers innovation and closed-loop supply chain pricing decisions for remanufacturing technologies under different government subsidies. In the recycling and remanufacturing segment, the impact of upgrading the level of remanufacturing technology on the remanufacturing of used products is innovatively presented. Two different subsidy mechanisms are introduced to provide a basis for government policy formulation. A CLSC decision-making model is developed to study how manufacturers adjust their remanufacturing technology investment strategies and retailers decide the optimal recycling price under different government subsidies. Then, the effects of different government subsidy levels on remanufacturing technology level, recycling price, supply chain profit, environment and social welfare are analysed through numerical simulations. Our goal is to enhance the theoretical research on sustainable CLSCs, but also to offer a position for the government's subsidy decisions, and to provide a theoretical basis to make remanufacturing technology input decisions, pricing decisions, and to promote the improvement of environmental and social welfare.

The rest of the paper is organized as follows: in Section 2, we afford a literature review of the article in terms of government subsidies and technological innovation. Section 3 presents our theoretical model to find answers to the research questions. Section 4 demonstrates the numerical analysis and affords some significant insights into present industry practices, and Section 5 comprises the conclusions of our work and probable future directions.

## 2 Review of literature

In this study, the literature relevant to this text was reviewed in two ways: government subsidies and technological innovation in closed-loop supply chains.

### 2.1 Government subsidies in CLSC

Many scholars conduct research on subsidies. some of which are subsidies per-unit cost of recycling or remanufactures products [9,10], the other part is a fixed cost proportion subsidy for recycling or remanufacturing processes [8,11,12]. In the literature on subsidising manufacturing, based on different subsidising entities, Cao et al. [13] examined the optimal production and pricing decisions of manufacturers selling remanufactured products and retailers selling new products in a dual-channel supply chain, taking into account a remanufacturing subsidy policy, i.e., subsidising the demand for remanufactured products, were investigated. Qiao & Su [7] examined the effect of government subsidies on the quantity of new products and remanufacturing, consumer surplus, social surplus, and the environment when the government used a subsidy policy to encourage remanufacturing by OEMs and independent remanufacturers based on the number of units of remanufactured products produced by both. Based on two vertically differentiated products, Hong et al. [14] investigates two subsidy policies, i.e., enterprise subsidy policy and consumer subsidy policy, and subsidises enterprises producing green products and consumers purchasing green products according to the quantity of the unit subsidy respectively, and derives the consumers' decision to purchase green or non-green products under different subsidies. Guo et al. [15] innovate a four-way evolutionary game model, which subsidises the production of enterprises adopting green behaviours or the consumption of consumers purchasing green products, to investigate what kind of government subsidies can effectively promote the sustainable development of the construction waste recycling system.

Zheng et al. [16] investigated the time decision of manufacturers to adopt green technologies under a policy of subsidising by fixed cost of equipment and a policy of subsidising by operating process. Bian et al. [17] investigates the incentive effect of environmental subsidies on the manufacturing industry to invest in emission reduction technologies by means of manufacturer's subsidy, which refers to the government sharing a portion of the cost of the manufacturer's environmental technology investment. Li et al. [18]investigated the effect of producer subsidy on the level of innovation in a two-tier supply chain structure where the government subsidises a certain percentage of the fixed cost of manufacturer innovation under different power structures. The above study points out that the government will subsidise companies to develop more environmentally friendly products for better environmental benefits. However, this type of supply chain innovation does not consider technological innovation in remanufacturing. Peng & Lin [19] Considers unit production subsidies and green technology investment subsidies to study how subsidy policies affect social welfare, manufacturers' profits and eco-innovation levels.

In the above studies, the focus is on describing the impact of subsidies on supply chain decision-making in the context of recycling subsidies and manufacturing subsidies, with subsidies based on a percentage of fixed investment and subsidies based on volume. Few of the government policies studied above have subsidies based on quantity and production costs. In addition, as remanufacturing is one of the main ways to save resources and protect the environment, the impact of subsidies on remanufacturing technological innovation is rarely considered in the above subsidy scenarios. Our study introduces remanufacturing technology innovation into a CLSC, considers two different types of subsidies, and discusses the impact of subsidies on technology innovation. Technological innovation also plays an important role in reducing carbon emissions and protecting the environment.

### 2.2 Technological innovation in the supply chain

A large number of scholars have conducted many high-level studies on technological innovation in supply chains. In some industries, manufacturers' pollutant emissions depend to a large extent on the quality of raw materials supplied by their suppliers. In such cases, suppliers' environmental innovation efforts are crucial for effective control of pollutant emissions. Based on the difference in innovation agents, some studies consider technological innovation investments by upstream supply chain members [11,[20], [21], [22]], some studies consider innovation conducted downstream of the supply [16,23]. Shen et al. [24] proposes product innovation by both upstream and downstream suppliers, with suppliers responsible for process innovation and manufacturers for product innovation; Dong et al. [25] considered how upstream innovations in the supply chain can affect downstream firms' innovations, and the study indicated that the impact of upstream innovations is positively moderated by the firms' absorptive capacity. In addition, Xiang & Xu [26] considered the impact of technological innovation of third-party Internet recycling platforms and big data marketing on the decision-making of closed-loop supply chain members.

The numerous studies above reflect that the subject of technological innovation is diverse and can be an upstream supplier, a manufacturer or a third-party platform. In a closed-loop supply chain consisting of manufacturers and retailers, we consider the manufacturer as the bearer of technological innovation and the dominant player in the CLSC. Xing et al. [27] considering that upstream start-up suppliers invest in quality innovation, financial constraints force firms to invest more in quality innovation, the study points out that the provision of investment-sharing contracts by manufacturers contributes to vertical R&D collaboration in supply chains. There are also scholars who have studied low-carbon innovation [19,28,29] and green innovation [20,30,31] in supply chains.

The previous section has considered innovations in technology such as green and carbon reduction, but little research has been done on manufacturers' remanufacturing technology innovations. Innovations in remanufacturing technology can enable manufacturers to find new profit growth points, and devote more attention to used waste products, the huge volume of which is the greatest resource for manufacturers. At the same time, it reduces the environmental pollution, which is also important for the improvement of human living environment. Although Shan et al. [32] mentioned remanufacturing technology innovation in their study, their study focused on whether manufacturers should engage in remanufacturing technology innovation. This paper investigates the effect of subsidies on the CLSC by analysing the level of innovation in remanufacturing technology.

## 3 Mathematical expressions and methods

### 3.1 Assumptions and model Description

**Fig. 1:**
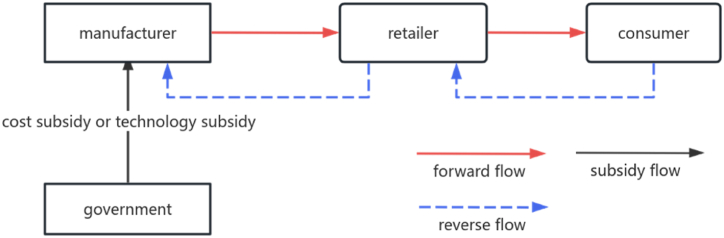
Closed loop supply chain structure.

In this paper, our research considers a single-period steady-state model, which can be interpreted as the mature period of the product life cycle. The market demand is assumed to be stable. Our study focuses on a CLSC comprising a manufacturer and a retailer. Based on extended manufacturer responsibility, manufacturers invest in remanufacturing technologies. Therefore, Stackelberg-game approach was adopted in this study, which considers the manufacturer as the leader in the game. The study analyses the effect of two subsidy mechanisms on the CLSC: a subsidy for remanufacturing costs and a subsidy for remanufacturing technology. The system structure is represented by Fig. 1, in which the manufacturer recycles used products through retailers. The manufacturer sells the product to the retailer at a wholesale price and is also responsible for recycling the used product from the retailer at a take-back price. Savaskan et al. [33] point out that retailer recycling is the best option because retailers are closer to consumers. As in the study of Zhou et al. [34], the study assumes that new and remanufactured products are of equal quality and price. By investing in remanufacturing technology, manufacturers can ensure that both new and remanufactured products achieve the same quality. Manufacturers wholesale new and remanufactured products to retailers at the same price. In order to encourage innovation in remanufacturing technology, governments subsidise manufacturers. It is imperative for governments to understand the effect of different subsidised incentives on remanufacturing activities.

Hypothesis 1 There are two ways for manufacturers to make materials for their final products: one is through purchasing new materials from upstream, and the other is through materials obtained by processing recycled products [26]. Manufacturers need to make a profit from the remanufacturing process, The manufacturing cost of a new product is higher than the manufacturing cost of a remanufactured product, i.e. $c_{r}<c_{n},c_{r}$ indicates the cost of the remanufactured product. Δ indicates the benefits obtained by the manufacturer through remanufacturing as a result of cost savings, $\Delta =c_{n}-c_{r}$. Manufacturers classify the quality of used products into two categories based on their level of remanufacturing technology: remanufactured products and recovered materials. Therefore, the study considers that manufacturers earn revenue per unit by selling materials recovered from the disposal of used products $p_{i}$. In addition, recycled products that can be remanufactured are more favourable to the manufacturer than the material from which the recycled products are sold, which has been expressed as $\Delta >p_{i}$.

Hypothesis 2 The study uses the level of remanufacturing technology to denote the level of t. And the cost to manufacturers of investing in remanufacturing technology is $\frac{\delta t^{2}}{2}$. δ is the investment cost per unit increase in the level of remanufacturing technology that a manufacturer pays when making an investment in remanufacturing technology. According to Han et al. [9], not all recovered products can be used for remanufacturing, and a portion of the used products can only be recycled for materials to be used in manufacture for cost savings or traded to other companies. The study considers that a manufacturer's investment in remanufacturing technology leads to a situation where it can use more of its recovered products for remanufacturing. When a manufacturer does not invest in remanufacturing technology, the proportion of used products that can be remanufactured is α, Then the proportion of recycled products that are used to sell materials is 1−α.

When a manufacturer makes an investment in remanufacturing technology, the proportion of used products that can be remanufactured from scrap is β, i.e. β=α+θt , Satisfied 0<θt<1−α, indicating that the state of the art will not allow all recycled products to be used for remanufacturing. θ representing the coefficient of increase in the proportion of used products that can be remanufactured as a result of improvements in remanufacturing technology. The proportion of recycled waste products that are used as raw materials for sale is 1−β .

Hypothesis 3 Manufacturer wholesales products at wholesale prices w, and pays transfer price b for recovery of used products from retailers. Retailers sell products at retail prices , and recover the product from the consumer at a recycled price $p_{c}$.In order to satisfy arbitrage behaviours in a CLSC, it needs to be satisfied $p>w>b>p_{c}$.

Hypothesis 4 According to Shan et al. [32], the study considers that investment in remanufacturing technology results in essentially the same new and remanufactured products, so consumers do not differentiate between the two products. Therefore, there are no competitive effects in the market for these two products. New and remanufactured products are perfect substitutes. According to the study of Huang et al. [35], the study considers that the demand function for the product produced by the manufacturer is continuous and deterministic and is assumed to be of the following form: D=q−ϕp, q represents the market size, ϕ represents the consumer price sensitivity factor. If ϕ relatively large, the increase in retail prices will have a more significant impact on market demand for products that are not essential to consumers, e.g. luxury goods. If ϕ relatively small, retail price increases do not have a significant impact on demand, e.g., flour, vegetables, etc. The total recycled quantity of used products is expressed as $=\varphi p_{c}$, φ indicates the sensitivity coefficient of consumers to the price of recycling. In a single period, it is unlikely that the consumer will be able to use all of the used products from the previous period for recycling and therefore satisfy <D.

Hypothesis 5 In order to simplify the expression of the two kinds of subsidies, the study considers that the two kinds of subsidies are expressed in a certain proportion. When the government subsidises the cost of a remanufactured product, it subsidises the manufacturer by a certain percentage of the cost per unit of remanufacturing; when the government subsidises investment in technological innovation in remanufacturing, it subsidises the manufacturer by a certain percentage of the amount of investment paid by the manufacturer.

Table 1 shows the notation and explanation in this paper.

**Table 1:** The table of notation and explanation.

Notation	Explanation
q	Market size
$c_{r}$	Production costs of remanufactured products
$c_{n}$	Production costs of new products
Δ	Unit cost savings of old products compared to new products
$p_{i}$	Revenue from the sale of materials obtained through the disposal of used products
δ	Coefficient of cost of investment in technological innovation
α	Proportion of old products available for remanufacturing without investment in technological innovation for remanufacturing
β	Proportion of old products available for remanufacturing with investment in remanufacturing technology innovation, β=α+θt, θ is the sensitivity factor of the level of technology to the proportion of used products that can be used for remanufacturing
φ	Consumer coefficient on recycling prices
ϕ	Consumer sensitivity coefficient to sales price
ρ	Discounted environmental impact per unit for remanufactured products relative to new products, 0<ρ≤1
$e_{1}$	Unit environmental impact of new products
$e_{2}$	The environmental benefit parameter, which indicates the extent to which recycling a unit of product brings environmental benefits to society.
s	Subsidy level
**Decision variables**	**Explanation**
t	Level of remanufacturing technology
*w*	Product Wholesale Prices
*b*	Transfer price for recycling of used and end-of-life products by manufacturers
*p*	Retail price
$p_{c}$	Recycling prices for retailers recycling used products
**Superscript**	**Explanation**
C∗	Optimal results under cost subsidies for remanufactured products
T∗	Optimal outcomes under subsidised investment costs for technological innovation

Based on the above assumptions, the study constructs a closed-loop supply chain model.

### 3.2 CLSC modelling with remanufacturing cost subsidies

In this section, the manufacturer, as the leader in the game, first decides on its own wholesale price, transfer price, and level of remanufacturing technology, and the retailer then sets its own retail and recycling prices based on the manufacturer's pricing decisions. In the process, the government subsidises the manufacturer with a percentage of the cost of the remanufactured product. Our model takes into account the impact of investment in remanufacturing technology, such that the proportion of products available for remanufacturing is increased by a rate determined θt by the remanufacturing technology.

In order to study the effect of remanufacturing technology innovation level and government subsidy, the research analyses the relationship between remanufacturing product cost subsidy and remanufacturing technology level, members' decision, and profit in the CLSC. The optimal model is represented as follows:

(1) $$\max_{w,b,t}\pi_{M}=wD-\beta Qc_{r}-\left(D-\beta Q\right)c_{n}+\left(1-\beta \right)Qp_{i}-bQ-\frac{\delta t^{2}}{2}+\beta Q\lambda c_{r}$$

(2) $$\max_{p_{c},p}\pi_{R}=\left(p-w\right)D+\left(b-p_{c}\right)Q$$

Equation (1) is the manufacturer's maximum profit function. The first term represents the manufacturer's revenue from wholesaling the product. The second term represents the cost of recovering the portion of the used product that can be used for remanufacturing, and the third term represents the cost of manufacturing the product using new materials when the recovered remanufactured product is insufficient to meet market demand. The fourth item represents the proceeds of disposal of the portion of recovered products that cannot be used for remanufacturing. The fifth item represents the cost of obtaining used products from retailers. The sixth item represents the cost of investing in remanufacturing technology, and the seventh term represents the manufacturer's income from government subsidies. Equation (2) is the maximum profit function for the retailer. The first term represents the retailer's revenue from the forward channel and the second term represents the retailer's revenue from the reverse channel.

By means of backward derivation, the study calculates the optimal decision for each member of the supply chain, Manufacturer's optimal decision:

$$w^{C*}=\frac{q+\phi c_{n}}{2\phi }$$

$$t^{C*}=\frac{\theta \varphi B_{1}\left(\alpha B_{1}+p_{i}\right)}{4\delta -\varphi \theta^{2}{B_{1}}^{2}}$$

$$b^{C*}=\frac{2\delta \left(\alpha B_{1}+p_{i}\right)}{4\delta -\theta^{2}\varphi {B_{1}}^{2}}$$

Optimal decision for retailers:

$$p^{C*}=\frac{3q+\phi c_{n}}{4\phi }$$

$${p_{c}}^{C*}=\frac{\delta \left(\alpha B_{1}+p_{i}\right)}{4\delta -\theta^{2}\varphi {B_{1}}^{2}}$$

Bringing optimal decision into Equations (1), (2). There are optimal profits for manufacturers and retailers, as Equation (3)and (4).

(3) $${\pi_{M}}^{C*}=\frac{{\left(q-\phi c_{n}\right)}^{2}}{8\phi }+\frac{4\varphi \delta^{2}{\left(\alpha B_{1}+p_{i}\right)}^{2}-\delta \theta^{2}\varphi^{2}{B_{1}}^{2}{\left(\alpha B_{1}+p_{i}\right)}^{2}}{2{\left(4\delta -\varphi \theta^{2}{B_{1}}^{2}\right)}^{2}}$$

(4) $${\pi_{R}}^{C*}=\frac{{\left(q-\phi c_{n}\right)}^{2}}{16\phi }+\frac{\varphi \delta^{2}{\left(\alpha B_{1}+p_{i}\right)}^{2}}{{\left(4\delta -\varphi \theta^{2}{B_{1}}^{2}\right)}^{2}}$$

In the equation (3)and (4) $B_{1}=\Delta +\lambda c_{r}-p_{i}$. Adding the optimal profits of the manufacturer and the retailer gives the total profit of the supply chain at this point, as Equation (5).

(5) $${\pi_{T}}^{C*}=\frac{3{\left(q-\phi c_{n}\right)}^{2}}{16\phi }+\frac{6\varphi \delta^{2}{\left(\alpha B_{1}+p_{i}\right)}^{2}-\delta \theta^{2}\varphi^{2}{B_{1}}^{2}{\left(\alpha B_{1}+p_{i}\right)}^{2}}{2{\left(4\delta -\varphi \theta^{2}{B_{1}}^{2}\right)}^{2}}$$

The study next analyses the closed-loop supply chain decision-making in the case of government subsidies for investment in remanufacturing technology innovation.

### 3.3 CLSC modelling under cost subsidies for remanufacturing technology innovation

In this subsection, the order of decision-making is the same as in the previous section. However, the type of government subsidy changes from a cost subsidy to a technology input subsidy. In this section, the study analyses the effect of investment in remanufacturing technology innovation and the whole supply chain in the case of government subsidies for remanufacturing technology innovation. The optimal model is as follows.

(6) $$\max_{w,b,t}\pi_{M}=wD-\beta Qc_{r}-\left(D-\beta Q\right)c_{n}+\left(1-\beta \right)Qp_{i}-bQ-\frac{\delta t^{2}}{2}+\lambda \frac{\delta t^{2}}{2}$$

(7) $$\max_{p_{c},p}\pi_{R}=\left(p-w\right)D+\left(b-p_{c}\right)Q$$

Equation (6) is a function of the maximum profit a manufacturer can make when making an investment in remanufacturing technology. The government will subsidise the manufacturer's investment in remanufacturing technology innovation on a pro rata basis. As a result of the remanufacturing technology innovation, the manufacturer will increase the percentage of remanufacturable used products. The retailer's profit function expression will not change, but the retailer's optimal profit will change with the manufacturer's innovation in remanufacturing technology and the government's subsidy for technological innovation, as Equation (7). By means of backward derivation, the study calculates the optimal decision for each member of the supply chain, manufacturer's optimal decision is:

$$w^{T*}=\frac{q+\phi c_{n}}{2\phi }$$

$$t^{T*}=\frac{\varphi B_{2}\theta \left(B_{2}\alpha +p_{i}\right)}{4\left(1-\lambda \right)\delta -\varphi {B_{2}}^{2}\theta^{2}}$$

$$b^{T*}=\frac{2\delta \left(1-\lambda \right)\left(B_{2}\alpha +p_{i}\right)}{4\left(1-\lambda \right)\delta -\varphi {B_{2}}^{2}\theta^{2}}$$

The retailer's optimal decision is:

$$p^{T*}=\frac{3q+\phi c_{n}}{4\phi }$$

$${p_{c}}^{T*}=\frac{\delta \left(1-\lambda \right)\left(B_{2}\alpha +p_{i}\right)}{4\left(1-\lambda \right)\delta -\varphi {B_{2}}^{2}\theta^{2}}$$

Bringing optimal decision into equations (6), (7), the research obtains the respective optimal profit, as Equation (8)and (9)

(8) $${\pi_{M}}^{T*}=\frac{{\left(q-\phi c_{n}\right)}^{2}}{8\phi }+\frac{4\varphi \delta^{2}{\left(\alpha B_{2}+p_{i}\right)}^{2}{\left(\lambda -1\right)}^{2}+\delta \left(\lambda -1\right)\theta^{2}\varphi^{2}{B_{2}}^{2}{\left(\alpha B_{2}+p_{i}\right)}^{2}}{2{\left(4\delta \left(\lambda -1\right)+\varphi \theta^{2}{B_{2}}^{2}\right)}^{2}}$$

(9) $${\pi_{R}}^{T*}=\frac{{\left(q-\phi c_{n}\right)}^{2}}{16\phi }+\frac{\varphi \delta^{2}{\left(\alpha B_{2}+p_{i}\right)}^{2}{\left(1-\lambda \right)}^{2}}{{\left(4\delta \left(\lambda -1\right)+\varphi \theta^{2}{B_{2}}^{2}\right)}^{2}}$$

In equation (8)and (9), $B_{2}=\Delta -p_{i}$. Adding the optimal profits of the manufacturer and the retailer gives the total profit of the CLSC under government subsidy of technology costs as Equation (10).

(10) $${\pi_{T}}^{T*}=\frac{3{\left(q-\phi c_{n}\right)}^{2}}{16\phi }+\frac{6\varphi \delta^{2}{\left(\alpha B_{2}+p_{i}\right)}^{2}{\left(\lambda -1\right)}^{2}+\delta \left(\lambda -1\right)\theta^{2}\varphi^{2}{B_{2}}^{2}{\left(\alpha B_{2}+p_{i}\right)}^{2}}{2{\left(4\delta \left(\lambda -1\right)+\varphi \theta^{2}{B_{2}}^{2}\right)}^{2}}$$

Here, the study haves solved the case of closed-loop supply chain decisions under different subsidies. However, government subsidies are often not aimed at the profitability of the supply chain, so the social benefits of different subsidies are examined next.

### 3.4 Social welfare

In this subdivision, the study further examines how governments set the optimal level of subsidies and subsidy methods for supply chains. The study consider that social welfare consists of the total profit of the CLSC, environmental impacts, and government expenditures. Based on the previous conclusions, the total profit of CLSC can be obtained. In this section, the study considers the effect of subsidies on the overall social welfare by expressing the social welfare as $\pi_{g}$,i.e. $\pi_{g}=\pi_{T}+E_{t}-s$.

He et al. [36] state that the use of recycled products rather than virgin materials reduces the environmental impact and that the environmental benefits of reproductions can be expressed in terms of a per unit environmental impact discount on top of the environmental benefits of new products. At this stage, the study considers the environmental benefits both in terms of the environmental impacts at the manufacturer's production stage and at the consumer's disposal stage. Therefore, the environmental benefits include two parts: the environmental benefits $E_{c}$ of recycling used products and the environmental benefits brought $E_{t}$ by remanufactured products, and the total environmental benefits of the whole CLSC are denoted by $E_{t}$ ,i.e. $E_{t}=E_{c}+E_{r}$.

$E_{r}$ indicates the environmental benefit to the manufacturer as a result of the production of the remanufactured product, where the environmental benefit per unit of remanufactured product produced is the difference in environmental benefit between the remanufactured product and the new product, provided that demand remains constant. ρ denotes the environmental discount factor for the remanufactured product, then the environmental benefit of producing the remanufactured product is $E_{r}=\beta Q\left(1-\rho \right)e_{1}$. $E_{c}$ denotes the environmental benefit to society from consumers using the product for recycling, i.e. $E_{c}=Qe_{2}$. Since lack of clarity on the specific use of recycled products that cannot be remanufactured, the environmental benefit of this component is assumed to be zero.

The study proceeds to discuss for the environmental benefits and social welfare in the two subsidy scenarios.

#### 3.4.1 Social benefits under cost subsidies for remanufactured products

The study haves already found the total profit of the CLSC in the case of such a subsidy. Based on the optimal decision made earlier, optimal demand and recovery can be derived, and therefore the two resulting environmental benefits, as Equation (11)and (12):

(11) $${E_{c}}^{C*}=\frac{e_{2}\varphi \delta \left(\alpha B_{1}+p_{i}\right)}{4\delta -\varphi {B_{1}}^{2}\theta^{2}}$$

(12) $${E_{r}}^{C*}=\frac{\left(4\alpha \delta +\varphi B_{1}\theta^{2}p_{i}\right)\varphi \delta \left(\alpha B_{1}+p_{i}\right)\left(1-\rho \right)e_{1}}{{\left(4\delta -\varphi {B_{1}}^{2}\theta^{2}\right)}^{2}}$$

Summing the two environmental benefits yields the total environmental benefit, as Equation (13):

(13) $${E_{t}}^{C*}=\frac{\left(4\alpha \delta +\varphi B_{1}\theta^{2}p_{i}\right)\varphi \delta \left(\alpha B_{1}+p_{i}\right)\left(1-\rho \right)e_{1}+e_{2}\varphi \delta \left(\alpha B_{1}+p_{i}\right)\left(4\delta -\varphi {B_{1}}^{2}\theta^{2}\right)}{{\left(4\delta -\varphi {B_{1}}^{2}\theta^{2}\right)}^{2}}$$

The total social welfare under the cost subsidy for remanufactured products is Equation (14):

(14) $${\pi_{g}}^{C*}={\pi_{T}}^{C*}+{E_{t}}^{C*}-s^{C*}$$

#### 3.4.2 Social welfare under subsidised cost of innovation in remanufacturing technologies

In next subsidy scenario, the study derives the environmental benefits of the different segments, as Equation (15)and(16):

(15) $${E_{c}}^{T*}=\frac{e_{2}\varphi \delta \left(1-\lambda \right)\left(\alpha B_{2}+p_{i}\right)}{4\left(1-\lambda \right)\delta -\varphi {B_{2}}^{2}\theta^{2}}$$

(16) $${E_{r}}^{T*}=\frac{\left(4\left(1-\lambda \right)\alpha \delta +\varphi B_{2}\theta^{2}p_{i}\right)\varphi \delta \left(1-\lambda \right)\left(\alpha B_{2}+p_{i}\right)\left(1-\rho \right)e_{1}}{{\left(4\left(1-\lambda \right)\delta -\varphi {B_{2}}^{2}\theta^{2}\right)}^{2}}$$

Similarly, the total environmental benefit of subsidising the cost of technological innovation is derived, as Equation (17):

(17) $${E_{t}}^{T*}=\frac{\left(4\left(1-\lambda \right)\alpha \delta +\varphi B_{2}\theta^{2}p_{i}\right)\varphi \delta \left(1-\lambda \right)\left(\alpha B_{2}+p_{i}\right)\left(1-\rho \right)e_{1}+e_{2}\varphi \delta \left(1-\lambda \right)\left(\alpha B_{2}+p_{i}\right)\left(4\left(1-\lambda \right)\delta -\varphi {B_{2}}^{2}\theta^{2}\right)}{{\left(4\left(1-\lambda \right)\delta -\varphi {B_{2}}^{2}\theta^{2}\right)}^{2}}$$

The total social welfare under the subsidised investment cost of the remanufacturing technology is ${\pi_{g}}^{T*}={\pi_{T}}^{T*}+{E_{t}}^{T*}-s^{T*}$.

The study has solved for the decision variables of closed-loop supply chains under different subsidy scenarios as well as profit, environmental benefits and social welfare. In order to visualize and compare the individual solutions, the study will next perform numerical simulations.

## 4 Results and numerical analysis

**Fig. 2:**
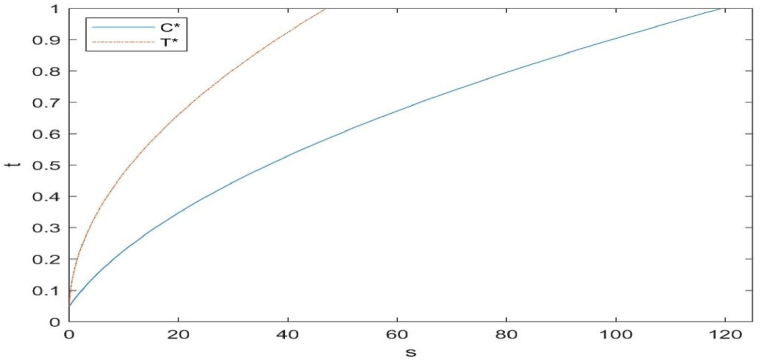
Effect of subsidy level on the level of remanufacturing technology.

**Fig. 3:**
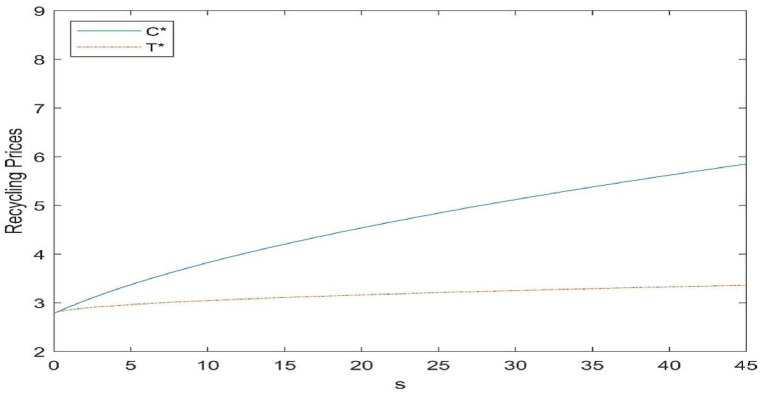
Effect of subsidy level on recovery price.

**Fig. 4:**
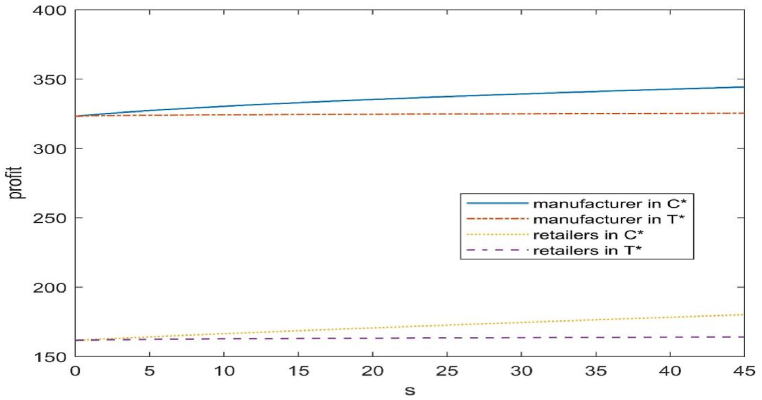
Impact of subsidies on the profitability of each member of the supply chain.

**Fig. 5:**
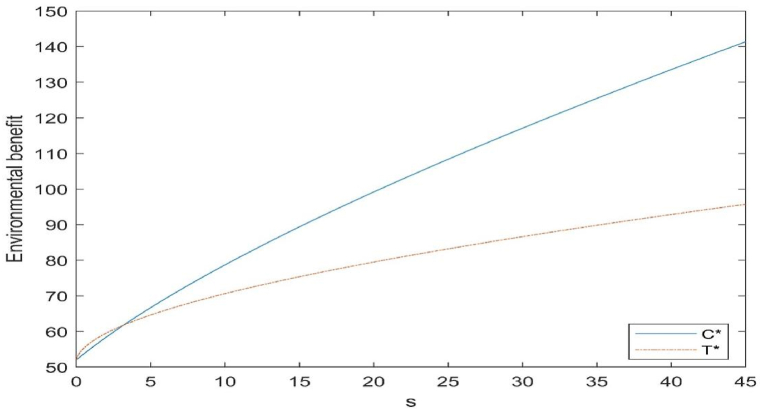
Impact of subsidy level on environmental benefits.

**Fig. 6:**
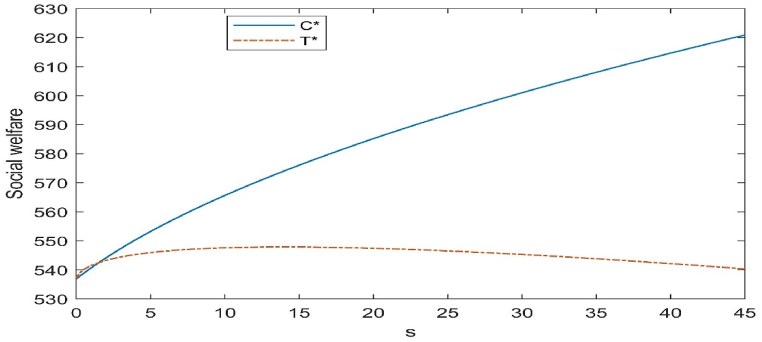
Impact of subsidy level on social welfare.

In this section, in order to better analyse the impact of the subsidy method and subsidy amount on the CLSC, numerical simulations are performed to derive theoretical results. The main objective of this paper is to provide a new research perspective and methodology on subsidies, which need to be parameterized according to the actual situation in a particular region of the globe. Based on previous studies [32], the study hypothesised that q=100, $c_{n}=50$, $c_{r}=35$; Δ=15, $p_{i}=10$, δ=100, α=0.2, θ=0.5, φ=0.7, $e_{1}=50$, $e_{2}=20$, ϕ=1, ρ=0.4. The optimal decision evolution trajectory is obtained, as shown in Fig. 2, Fig. 3, Fig. 4, Fig. 5, Fig. 6.

### 4.1 Effect of subsidies on CLSC

The study first compares the effects of subsidies on the level of remanufacturing technology, recycling prices, and the profitability of each member of the supply chain through numerical simulations.

#### 4.1.1 Effect of subsidies on the level of remanufacturing technology

As can be seen from Fig. 2, the two types of subsidies are positively correlated with manufacturers' remanufacturing technology levels, and in the absence of subsidies, manufacturers are willing to invest in remanufacturing technology innovation levels on their own. From the perspective of subsidy amount, government subsidies on remanufacturing technology costs are more effective in motivating manufacturers to carry out remanufacturing technology innovation. According to our hypothesis, if the manufacturer's optimal technology level is 1, based on the data from our numerical simulation, the numerical simulation can derive the maximum cost of different government subsidies, which is 45 when the government decides to subsidise the investment cost of remanufacturing technology, and 119 when the government decides to subsidise based on the cost of remanufacturing per unit and the number of used products that can be used for remanufacturing.

The conclusion can be drawn that from the point of view of remanufacturing technology innovation, if the government wants to stimulate social enterprises to invest in remanufacturing technology innovation at a lower cost, it would be more effective to use direct subsidies on the cost of investment in remanufacturing technology. In addition, there exists a subsidy ceiling for the two kinds of subsidies, and reaching the subsidy ceiling, the two kinds of government subsidies will not be in the impact on the level of remanufacturing technology.

#### 4.1.2 Effect of subsidies on recycling prices

Recycling price refers to the cost to the retailer of recycling the product from the consumer, i.e. the amount received by the consumer. A higher amount received by the consumer means that more consumers are willing to recycle their used products. Since the price of recycling is directly related to the quantity of recycling, the effect of the two subsidies on the price of recycling indirectly reflects the effect of the two subsidies on the quantity of recycling. In order to better compare the advantages and disadvantages of the two types of subsidies, the study will consider the impact of the two types of subsidies on the CLSC and the society within the subsidy amount of 45.

As can be seen in Fig. 3, there is a positive correlation between the two types of subsidies and the price of recycling. When the amount of subsidy is the same, when the government subsidises the unit cost of the remanufactured product, the consumer receives more and recovers more, while when the government subsidises the investment cost of the remanufacturing technology innovation, the consumer receives less. Through the transformation of the graph we can see, no matter which form of subsidies, will be reflected in the recycling price, the results of subsidies will not only make manufacturers more willing to invest in remanufacturing technology, will also make the recycling quantity to a certain degree of enhancement.

Compared with Fig. 2, it is easy to find that under the same amount of subsidy, when the government invests in remanufacturing technology, the manufacturer will spend more energy on remanufacturing technology innovation to advance the level of remanufacturing technology in order to attain the subsidy and do less to improve the recycling volume of used products, which will result in the manufacturer's total revenue not necessarily being the same although the level of remanufacturing technology is higher, as we discuss in the next section; when the government subsidises the cost of remanufactured products, the manufacturer will increase the recycling price of used products in order to obtain the subsidy revenue.

The conclusion can be drawn that subsidising the cost of remanufactured products would be a more effective approach for governments, where recycling quantities are targeted, and that consumers are more inclined to favour such a subsidy.

#### 4.1.3 Effect of subsidies on the profitability of supply chain members

From Proposition 1 and Fig. 2, Fig. 3, it can be seen that the main way for manufacturers to improve their profits, i.e. to reduce their own production costs, is mainly from two aspects: on the one hand, to advance the level of remanufacturing technology, so that more used products can be used for remanufacturing under the premise of a certain amount of recycling to reduce their own manufacturing costs; then again, to recycle more used products, so that under a certain level of remanufacturing technology, to make more products for sale by the remanufacture of used products in order to reduce their own manufacturing costs.

As can be seen in Fig. 4, both types of subsidies are positively correlated with the profits of each member. In the CLSC there is a free-rider phenomenon, the government in the case of subsidies to manufacturers, manufacturers in order to improve the profit at the same time, the retailer's profits are also increased; in the same amount of subsidies, subsidies to the cost of remanufactured products will bring greater benefits to the manufacturer and the retailer, in the case of the subsidy, the number of recycling is large, the level of remanufacturing technology is relatively low, but the benefits obtained are greater. It also suggests that an increase in the amount of recycling increases the profitability of each member of the CLSC more than an increase in the level of remanufacturing technology increases the profitability of each member.

### 4.2 Effect of subsidies on environmental benefits

We divide the environment into two components: the environmental benefits of recycling used products and the environmental benefits of remanufacturing used products to reduce the use of raw materials. The environmental benefits of recycling are directly related to the amount of recycled products. The remanufacturing of used products is directly related to the level of remanufacturing technology.

As can be seen in Fig. 5, the two types of subsidies are positively correlated with environmental benefits, and there is a threshold (i.e. 3.32, intersecting points in Fig. 5) intersection between the two types of subsidies. When the amount of subsidy is lower than the threshold, subsidising the cost of investment in remanufacturing technology leads to more environmental benefits from the CLSC, while when the amount of subsidy is higher than the threshold, subsidising the cost of remanufactured products leads to more environmental benefits from the closed-loop supply chain. It shows that when the amount of subsidy is relatively small, the number of recycling under the two types of subsidy is similar, and subsidising the investment cost of the level of remanufacturing technology leads to a significant increase in the level of remanufacturing by the manufacturer, so that more waste products can be remanufactured, and since the environmental benefit caused by remanufacturing per unit of waste product is higher than that caused by recycling per unit of waste product, when the government budget is small, that type of subsidy will produce greater environmental benefits. On the contrary, when the amount of subsidy is higher, the level of remanufacturing technology under the subsidy on the cost of remanufactured products will not only gradually improve, but also the quantity of recycled products will become more, and the proportion of remanufactured products will be increased on the basis of recycling more used products, therefore, when the government budget is higher, the subsidy on the cost of remanufactured products can bring greater environmental benefits.

### 4.3 Effect of subsidies on social welfare

The study considers that social welfare is composed of the total profit of the CLSC, the cost of government subsidies, and environmental effects. The main purpose of government policies is also to maximise social welfare. Fig. 6 shows that when the government subsidises the cost of remanufactured products, the social welfare displays an increasing trend, while when the government subsidises the investment cost of remanufacturing innovation technology, the social welfare displays an increasing and then decreasing trend. There is a threshold (i.e. 1.69, intersecting points in Fig. 6) intersection of the two subsidies. When the government subsidy is beneath the threshold, the social welfare of subsidising the cost of remanufacturing technology is higher and more effective, mainly due to the increase in the level of technology. When the government subsidy is above the threshold, the social welfare of subsidising the cost of remanufactured products is higher and more effective, mainly due to the increase in the number of recoveries.

As can be seen in Fig. 6, the increase in unit social welfare for both subsidies slows down as the subsidy increases, and to a greater extent in the case of subsidising the cost of the level of remanufacturing technology. The intersection of the two subsidies in the graph may be different under different assignments, depending on the actual situation. In addition, the conclusion shows that there is an optimal level of subsidy for subsidising the cost of innovation in remanufacturing technology. That is, if the subsidy amount is less than the optimal subsidy amount, the more the subsidy amount, the higher the social welfare, if the subsidy amount is higher than the optimal subsidy level, the less the subsidy amount, the higher the social welfare effect.

## 5 Conclusions and discussion

In this study, a CLSC consisting of retailers and manufacturers is constructed. This paper is modelled on Stackelberg game theory. The analysis is carried out through numerical simulations to investigate the impact of different subsidies on investment in remanufacturing technologies, pricing decisions, firm revenues and social welfare. The conclusions are summarised as follows:

• In both subsidised scenarios, the increase in retailer profits is due to an increase in recycling volume and recycling price.Under the subsidy for the cost of remanufactured products, the consumers are more willing to use the waste products for recycling. When the government subsidises the cost of remanufactured products, the combined effect of the two is greater, and manufacturers can earn higher profits.
• The government can achieve the effect of increasing the level of innovation in remanufacturing technology through both types of subsidies. In order to minimize the subsidy investment, the government can subsidise the investment in remanufacturing technology innovation, so that the manufacturer's remanufacturing technology level can reach the highest level with less money. In addition, for the purpose of improving the level of remanufacturing technology, there is a subsidy ceiling for both types of subsidies.
• Taking the maximisation of environmental benefits or social welfare as the purpose of subsidies, it can be concluded that: when the amount of government subsidies is below the threshold, subsidies for investment in innovation in remanufacturing technology can lead to higher environmental benefits or social welfare; when the amount of government subsidies is above the threshold, subsidies for the cost of remanufactured products can lead to higher environmental benefits or social welfare. In addition, the social welfare of subsidising innovative investment in remanufacturing technology has a maximum value with respect to the amount of subsidy.

From a theoretical perspective, this study introduces remanufacturing technological innovation into the supply chain, considers the impact of government subsidies on the level of manufacturers' remanufacturing technology and pricing decisions in the closed-loop supply chain, and enriches the research on subsidies and remanufacturing in the closed-loop supply chain. In addition, the study provides new anchoring criteria for government subsidies from a practical perspective. It shows the government how a policy of subsidising the remanufacturing segment of the supply chain will affect consumers, the environment, social welfare, etc.

In the future, further research can be carried out in the following areas. First, future research can consider direct recycling of used products by manufacturers. Second, the government's publicity will also affect the quantity of recycling and the demand for recycling, and there are many factors that have an impact on it, which we can take this into account in the future. Finally, the conclusion of this paper is obtained only through static games, however different game scenarios between manufacturers and retailers can be further researched.

## Funding statements

This research was funded by Shandong Province Higher Education Philosophy and Social Science Research Project. The grant is (2024ZSMS092).

## Data availability statement

No data was used for the research described in the article.

## Additional information

No additional information is available for this paper.

## CRediT authorship contribution statement

**Peng Wan:** Writing – original draft, Supervision, Project administration. **Zhiyuan Xie:** Writing – review & editing, Methodology, Conceptualization.

## Declaration of competing interest

The authors declare that they have no known competing financial interests or personal relationships that could have appeared to influence the work reported in this paper.
